# mHealth–Supported Perioperative Care for Family Caregivers of Children Undergoing Tonsillectomy and/or Adenoidectomy (TONAPP): Randomized Controlled Trial

**DOI:** 10.2196/93989

**Published:** 2026-09-01

**Authors:** Raffaella Dobrina, Chiara De Vita, Laura Brunelli, Giulia Galvani, Margherita Dal Cin, Manuela Giangreco, Milena Ciampechini, Silvana Schreiber, Giada Ferrari, Paola Di Rocco, Sara Zaccariotto, Maria Lucrezia Saija, Ilaria del Giorno, Sara Zanchiello, Anja Starec, Andrea Cassone

**Affiliations:** 1 Institute for Maternal and Child Health - IRCCS “Burlo Garofolo”, via dell’Istria 65/1, Trieste, Friuli Venezia Giulia, 34137, Italy, +39 040 3785739; 2 Area Science Park, Trieste, Friuli Venezia Giulia, Italy; 3 Department of Medical, Surgical and Health Science, University of Udine, Udine, Italy; 4 Department of Health Prevention, Azienda Sanitaria Universitaria Giuliano isontina, Trieste, Friuli Venezia Giulia, Italy; 5 Central Directorate for Health, Social Policies and Disability, Trieste, Autonomous Region of Friuli Venezia Giulia, Italy

**Keywords:** family-centered care, mHealth, app, pediatric surgery, caregiver education, caregiver information, anxiety

## Abstract

**Background:**

Pediatric ear, nose, and throat (ENT) surgery is common, but generates perioperative anxiety for caregivers and distress in children. Limited time for perioperative education and reliance on unverified online information can reduce family preparedness and increase stress. Few studies have evaluated co-designed mobile health (mHealth) apps to support and engage families in the perioperative ENT journey.

**Objective:**

This study aimed to compare caregiver anxiety between an mHealth app-supported care pathway and standard supportive and educational care alone in the perioperative ENT context. Secondary objectives explored between-group differences in caregiver anxiety at follow-up, family preparation, child distress, and social-impact indicators.

**Methods:**

A 2-arm, parallel-group, open-label randomized controlled trial (RCT) enrolled caregivers of children undergoing ENT surgery (tonsillectomy, adenoidectomy, tympanostomy tube insertion). The intervention was an mHealth app co-designed through a user-centered participatory approach and developed following Schnall and colleagues’ Information Systems Research Framework, with content based on caregivers’ informational needs. RCT participants were recruited at the hospital during their presurgery visit, when a health care provider introduced the study and provided instructions on how to use the app. No additional human support was scheduled thereafter. A sample size of 180 participants (90 per group) was estimated to detect the expected between-group difference in caregiver anxiety. Participants were randomly assigned in a 1:1 ratio to app use or standard care alone. The primary outcome was the between-group difference in caregiver state anxiety (State-Trait Anxiety Inventory [STAI-Y]). Secondary outcomes included between-group differences in child distress (modified version of the Yale Preoperative Anxiety Scale [mYPAS]), child preparation for surgery, family preparation for hospital admission and surgery, and social impact indicators. Outcomes were assessed online through questionnaires, which included both self-reported measures and evaluations completed by a nurse on the day of surgery. App engagement metrics were also collected. Reporting followed the CONSORT-EHEALTH (Consolidated Standards of Reporting Trials of Electronic and Mobile Health Applications and Online Telehealth) guidelines.

**Results:**

The study enrolled 227 caregivers, with 111 allocated to the control group (CG) and 116 to the experimental group (EG), achieving the target sample size. No statistically significant differences were observed between the groups for the primary or secondary outcomes (all *P*>.05). In the EG, 75% (n=87) of the participants accessed at least 1 item of in-app content. Higher baseline anxiety was linked to lower app use (ρ=–0.22, 95% CI –0.39 to –0.04; *P*=.02), while greater use was linked to lower child distress (ρ=–0.23, 95% CI –0.40 to –0.04; *P*=.02).

**Conclusions:**

Although the hypotheses were not confirmed, these findings provide valuable insights for future perioperative mHealth research. The lack of effectiveness may reflect limited exposure to the intervention, outcome selection and timing, and contextual factors such as caregivers’ independent information-seeking. These findings support a greater focus on implementation processes and on identifying the caregivers most likely to benefit from mHealth-supported education.

## Introduction

### Background

Pediatric ear, nose, and throat (ENT) surgery has undergone substantial advances over recent decades, with the introduction of innovative surgical techniques and standardized clinical pathways. In this context, elective procedures, such as tonsillectomy and/or adenoidectomy, with or without tympanostomy tube insertion, remain among the most frequently performed surgical interventions in children [1-5].

These procedures are typically delivered in high-pressure clinical settings characterized by short hospital stays and limited time for structured education. For children and their caregivers, the perioperative pathway is often unfamiliar and information-dense, leaving little opportunity to adequately address the different phases of the process, potential intraoperative risks, and common postoperative complications [6]. Postoperative sequelae may include pain, bleeding, nausea, vomiting, infection, swallowing difficulties, and prolonged discomfort after discharge. These complications can reduce oral intake, lead to dehydration, disrupt sleep, and, in more severe cases, result in hospital readmission and repeated school absences with potential academic consequences, thus placing considerable emotional and organizational demands and burden on families [5-8]. In addition, emotional factors play a key role in shaping perioperative outcomes in children, influencing both preoperative preparation and postoperative management and recovery [9]. In particular, perioperative anxiety has been consistently associated with adverse postoperative outcomes. Evidence shows that children who experience higher levels of anxiety before surgery are more likely to report increased postoperative pain and face a higher risk of complications [10,11]. Elevated preoperative anxiety in young children is also associated with increased sleep disturbances and higher levels of postoperative anxiety during recovery [10]. From a behavioral perspective, children may also exhibit problematic behavioral changes and temporary limitations in daily activities in the immediate postoperative period following ENT surgery [12].

Consistent with the family-centered approach, which considers family caregivers as key partners throughout the perioperative pathway, parental emotional responses represent another critical component of the pediatric perioperative experience. In fact, parental anxiety has been consistently identified as a significant predictor of children’s anxiety prior to surgery [13] and appears to play a decisive role in shaping postoperative outcomes. Several studies have shown that higher levels of preoperative parental anxiety are associated with increased postoperative pain in children, a longer duration of analgesic use, and a higher number of analgesic administrations following tonsillectomy with or without adenoidectomy [4]. Similarly, children undergoing adenotonsillectomy whose parents report elevated anxiety levels have been found to experience higher rates of postoperative pain and complications [5]. Therefore, addressing parental anxiety before and during hospitalization is crucial not only to improve children’s postoperative recovery but also to reduce emotional distress in both children and caregivers throughout the perioperative process. Interventions aimed at supporting parents’ coping strategies and self-efficacy, including the provision of clear, timely, and tailored information, have been recognized as particularly relevant in pediatric surgical care [14-17].

Considering the complexity of the ENT perioperative process and its multiple clinical, emotional, and organizational implications, the provision of effective preoperative and postoperative education for both children and their family caregivers represents an essential component of care. In this context, parents’ health literacy (HL), defined as “the knowledge, motivation, and competencies to access, understand, appraise, and apply health information in order to take judgements and take decisions in everyday life concerning health care, disease prevention and health promotion” [18], plays a pivotal role in how families navigate the perioperative surgical pathway. Evidence indicates that a substantial proportion of parents and caregivers of pediatric surgical patients have limited HL, which may negatively affect their understanding of surgical conditions, perceived risks and benefits, adherence to preoperative and postoperative instructions, and the quality of informed consent, while also contributing to higher levels of anxiety during surgical consultations [19-21]. These difficulties may be exacerbated by time constraints during consultations and the use of medical terminology that caregivers may find difficult to understand or feel uncomfortable questioning [22].

Moreover, the literature has revealed that parents tend to self-educate by relying on available online health care information, quickly seeking answers to their questions directly and anonymously from home [23,24]. These practices expose them to the risk of accessing information that, coming from very heterogeneous sources, is unreliable and not scientifically validated [25].

Within this framework, addressing these educational and communicative challenges requires structured perioperative preparation strategies that support both children and families throughout the ENT surgical pathway. Evidence suggests that nonpharmacological and digital interventions, including educational and immersive approaches aimed at familiarizing children with the hospital environment, equipment, and planned procedures, can reduce anxiety and emotional distress in pediatric surgical patients, particularly when integrated into perioperative preparation [15-17].

Building on these findings, perioperative digital interventions have been increasingly implemented across pediatric surgery. These interventions range from apps to text-messaging systems that support education, symptom monitoring, and reminders for medications or follow-up appointments. Overall, the available evidence suggests potential benefits in outcomes such as follow-up adherence, postoperative monitoring, and user satisfaction, although study quality is variable and several trials show limitations (eg, inadequate controls, lack of blinding, and unequal attention to the experimental group [EG] and control group [CG]) [26].

Evidence syntheses also indicate that digital and distraction-based strategies, particularly virtual reality and other interactive formats, can reduce children’s preoperative anxiety and may improve immediate perioperative outcomes, supporting their broader adoption in practice [15-17,27]. However, much of the literature focuses on single time windows (eg, preoperative anxiety reduction, postoperative follow-up or monitoring) rather than supporting families across the full perioperative pathway in a coherent way [15,26]. Recent evidence indicates that perioperative programs integrating multiple interventions across different time points, rather than focusing on a single moment of care, may achieve greater effectiveness in reducing parental and child distress [28].

Moreover, parental anxiety has been recognized as a relevant and modifiable target, with reviews describing multiple approaches (eg, education, audiovisual tools, preparation programs, and parental presence strategies), but with heterogeneity in content and delivery and limited clarity on which components work best for which families [28]. Furthermore, prior randomized controlled trials (RCTs) and meta-analyses include heterogeneous surgical populations, limiting conclusions specific to tonsillectomy and/or adenoidectomy with or without tympanostomy tube insertion [15].

Importantly, parents report valuing information provided directly by the hospital, suggesting that institution-endorsed digital tools may be particularly acceptable and useful for addressing practical issues such as pain management and fasting discussions at home [29]. Within this framework, there is a need for a condition-specific, hospital-based mobile health (mHealth) intervention designed to support family caregivers across milestones of the pediatric ENT perioperative process, using a user-centered participatory approach, with outcomes assessed through a rigorous trial design [30].

### The Study: Rationale and Objectives

Considering the gaps in the literature, the aim of the current study was to evaluate the effectiveness of an mHealth app in supporting family caregivers of pediatric patients undergoing tonsillectomy and/or adenoidectomy with or without tympanostomy tube insertion, compared with supportive and educational methods related to standard care, in an RCT. Building on existing evidence, the present study focuses on the implementation of an mHealth system tailored to a specific pediatric surgical context and a clearly defined group of end users, considering the multiple phases of the ENT perioperative process. The mHealth app used in the intervention was a custom-designed, disease-specific, accessible tool, capable of providing validated scientific information, complying with current safety and privacy regulations, and designed to accompany children and their family caregivers throughout the perioperative process [31].

In line with the general aim, we intended to pursue 2 objectives. The primary objective was to explore the effect of using the mHealth app on the level of anxiety experienced by caregivers compared with standard care. The secondary objectives of the RCT were to evaluate the impact of using the mHealth app, compared with standard care, on 3 educational-emotional-organizational aspects, including (1) child preparation for hospitalization and surgery, (2) child distress before surgery, and (3) social impact such as postdischarge management. The study is based on the hypothesis that the possibility of using the mHealth app could help both reduce caregivers’ state anxiety (primary outcome) and improve child preparation for hospitalization and surgery, child distress, and social impact related to postdischarge management at home (secondary outcomes).

## Methods

### Study Design and Setting

A full description of the design and methods used is contained in the study protocol [32]. The study used a 2-arm, parallel, open-label RCT involving family caregivers of pediatric patients aged between 2 and 10 years, who were scheduled for tonsillectomy and/or adenoidectomy with or without insertion of a tympanostomy tube. The study was conducted within a superiority framework, aiming to determine whether the EG yields better outcomes compared to the CG.

Blinding of participants, nurses, and researchers involved in the study was not feasible due to the nature of the intervention. Caregivers allocated to the EG were required to download and actively use the mHealth app and could seek support from nurses throughout the perioperative pathway, whereas those in the CG received standard care alone. In addition, nurses and researchers were involved in participant enrollment and data collection activities within the clinical setting, and app use could become apparent during routine clinical interactions, making concealment of group allocation impracticable.

The monocentric study was conducted at a 136-bed maternal and child health hospital (MCHH) in Northern Italy, where approximately 450 tonsillectomies or adeno-tonsillectomies are performed each year. The participant enrollment process, which took place in the consulting rooms of the hospital’s pediatric ENT surgery department, began in December 2022 and concluded in December 2024. The random allocation sequence was generated independently by the hospital’s expert statistician from the Clinical Epidemiology and Public Health Research Unit using computer-generated randomization with a block size of 4. Allocation concealment was ensured through sequentially numbered, opaque, sealed envelopes. Personnel involved in participant enrollment did not have access to the allocation sequence.

Nursing staff responsible for enrollment were in charge of providing eligible family caregivers with information about the study and obtaining signed informed consent from those wishing to participate in the RCT. Participation was voluntary, and caregivers could withdraw from the study at any time. The nurses, previously adequately trained in the mHealth app content, features, and functionalities, were also responsible for instructing the EG caregivers both on how to download the app onto their smartphones or tablets and on how to use its contents and functionalities. During this training session, the nurses also showed the EG participants a 3-minute video presentation of the app on a tablet.

This trial is reported in accordance with the CONSORT-EHEALTH (Consolidated Standards of Reporting Trials of Electronic and Mobile Health Applications and Online Telehealth) checklist (Checklist 1) [33].

### Participants

Eligible participants were caregivers of children aged 2 to 10 years scheduled for tonsillectomy and/or adenoidectomy, with or without tympanostomy tube insertion. Caregivers were required to be able to communicate in spoken and written Italian and to have access to a smartphone and an internet connection. Exclusion criteria included cognitive or visual impairment in caregivers, cognitive impairment and/or chronic pain conditions in the child, previous surgery within the last month, and lack of previous experience with smartphone apps among caregivers. A formal assessment of reading comprehension was not performed during recruitment. However, eligibility was verified through direct interaction with caregivers, including confirmation of their ability to communicate in spoken and written Italian. HL was assessed as a study variable rather than used as an eligibility criterion, as the aim was to evaluate the intervention in a caregiver population representative of routine clinical practice.

After enrollment, participants were recruited in person at the hospital, on the day of their pre-surgery visit. Participants were not required to pay to access the app. Participants were randomly assigned in a 1:1 ratio by opening a sequentially numbered opaque and sealed envelope to either the EG, which received access to the mHealth app in addition to standard care, or the CG, which received standard care only. Standard care comprised oral information and education on the ENT perioperative pathway, supplemented by printed brochures provided by physicians and nurses during preoperative visits and throughout the hospitalization period.

### Intervention Description

The intervention tested in this study was a purely app-based intervention. It allowed participants in the EG to access and use an mHealth app on their smartphones to acquire information and education about the ENT perioperative process related to 2 specific surgical procedures, namely tonsillectomy and/or adenoidectomy with or without insertion of a tympanostomy, in addition to receiving information and education on standard perioperative care provided by the ward’s health care professionals. The EG was allowed to access the mHealth app from the day of the presurgery visit or preadmission consultation—when the enrollment took place—until the day of the scheduled follow-up visit, about 7 days after surgery. There was no mandatory minimum number of content views.

The contents, features, and functionalities of the mHealth app have been designed and developed by adopting a user-centered participatory design approach, in line with the Information System Research (ISR) framework [34], through the exploration of the informational or educational needs and preferences of both family caregivers (ie, primary end-users) and health care providers (ie, secondary end users) toward the mHealth app itself. A detailed description of the participatory design approach has already been published [30]. This co-design and codevelopment process involved the MCHH, which hosted the RCT and a public research institution, both situated in northern Italy.

Regarding content, the mHealth app contained 116 PDF documents covering the following six different thematic areas corresponding to 6 key informational or educational moments in the ENT perioperative process identified by primary and secondary end users who were directly involved in the participatory development process of the mHealth app: (1) the first inpatient ENT surgical consultation; (2) the phone call from the ENT surgical planning office to the family caregiver to communicate the date of the planned surgery and a list of necessary documents to bring on the day of preadmission consultations; (3) the day of preadmission consultations when caregivers are responsible for delivering the documents necessary for hospital admission and nursing, during which the anesthesiological and surgical consultations and examinations are also conducted; (4) the day of the family’s admission to the hospital and where the surgery is performed; (5) discharge from the hospital and preparation for postoperative care at home, generally on the same day as the surgery (in the absence of complications); and (6) the follow-up visit in the hospital which takes place approximately 7 days after surgery. The app’s contents were validated by the research team and the MCHH Scientific Director. Screenshots of the app have been published elsewhere [30].

The app’s content was delivered in accordance with a timing principle, ensuring that information relevant to the specific stage in the ENT perioperative process was presented to the caregiver at the appropriate moment. In particular, depending on the child’s stage in the perioperative process (eg, the day of preadmission consultation, the day of hospital admission and surgery, or postoperative recovery at home), the relevant content was suggested to the family caregiver through pop-up notifications as newly available content to consult within the app.

All the proposed content included validated medical information and reliable scientific references. All documents were provided in Italian and in text format. Depending on the specific topic covered, documents could include images, hyperlinks, graphical representations, and/or links to in-depth videos designed to facilitate caregivers’ understanding. To access specific information content, caregivers had to open the corresponding PDF document in the app. Although the educational content was designed to be conveyed to the study participants progressively, reflecting the evolution of the ENT perioperative process, family caregivers still had access to all the documents throughout the period between enrollment and the follow-up visit, since they were available to end users at any time in a dedicated section of the mHealth app called Library in a consumable format and in line with the “just in time” principle [35].

As anticipated in the *Study Design and Setting* section, both participants in the EG and CG benefited from the supportive educational methods of standard care provided by health care personnel (ie, physicians and nurses), both orally and through the delivery of printed brochures. This education on the ENT perioperative process was comparable to that conveyed to the EG via the mHealth app, including content on strategies for preparing children for the experience of hospitalization, the specific surgical procedure selected, the type of anesthesia chosen, or managing postoperative pain in children at home after discharge. During the RCT, neither the EG nor the CG was prevented from seeking and using further information from books or other print materials, consulting other health care professionals, or web or social media sources. This approach was intentionally adopted to reflect routine clinical practice, where caregivers are exposed to and may actively seek information from multiple sources. The intervention was therefore evaluated as an adjunct to usual information-seeking behaviors rather than as a replacement for them.

### Measures Used to Evaluate the Outcomes

The measures were taken at 4 different times, including T0 (ie, the enrollment), T1 (ie, the day of preadmission consultations), T2 (ie, the day when the surgery was performed), and T3 (ie, the follow-up visit). The timing, administration, and scoring methods for each measure are indicated below.

#### Sociodemographic Questionnaire

A sociodemographic questionnaire was self-assessed by participants at T0 to collect information on the family caregiver’s age, gender, education level, occupational status, type of relationship with the child (ie, mother, father, other guardian), possible employment as a health professional, and any previous experience in caring for others with health problems. The questionnaire also asked caregivers about the child’s age and sex, the surgical procedure planned during the current hospitalization, and any previous surgical experiences the child had undergone.

#### HL

Caregivers’ level of HL was assessed at T0 using the 16-item self-assessment version of the European Health Literacy Survey Questionnaire (HLS-EU-Q16) [36]. This tool provides 5 possible answers on a 4-point Likert scale ranging from 1 (very difficult) to 4 (very easy), with a fifth possible answer equivalent to “I don’t know.” The expected score range is 0‐16 and, depending on the total score obtained, 3 levels of HL can be distinguished, that is inadequate (0‐8), problematic (9–12), and sufficient HL (13–16).

#### Caregiver’s Preparation for Hospital Admission and Surgery

At T1, administrative nurses assessed caregivers’ preparation for hospital admission and surgery using a checklist to verify the completeness of the necessary documentation provided by parents when they arrived at the hospital. The number of missing documents (eg, identity card or health care card) was recorded.

#### Child’s Preparation for Surgery

At T2, a nurse in the surgical department assessed whether all the instructions provided by the staff for standard child surgical preparation had been followed, including proper hygiene, fasting, and not wearing nail polish or jewelry.

#### Caregiver’s Anxiety

Caregivers’ anxiety was evaluated using the Italian version of the State-Trait Anxiety Inventory questionnaire [37], consisting of 2 self-report scales for assessing state anxiety and trait anxiety, respectively: (1) the STAI Form Y-1 (STAI-S), including 20 statements aimed at measuring how the family caregiver feels “now, at this moment” on a 4-point Likert scale ranging from “not at all” to “very much”, and (2) the STAI Form Y-2 (STAI-T), comprising a further 20 statements aimed at assessing how the participant feels “generally” on a 4-point Likert scale ranging from “almost never” to “almost always.” The total score ranged from 20 to 80 points, and lower scores corresponded to higher levels of trait and state anxiety. Family caregivers were administered both scales at T0 to establish baseline levels of state and trait anxiety. The STAI-S scale was then further administered to caregivers in the hospital’s ENT surgical department at both T2, before the surgery was performed, and T3, at the follow-up visit, approximately 7 days after surgery.

#### Child’s Distress

To assess the child’s distress at T2, a modified version of the Yale Preoperative Anxiety Scale (m-YPAS) [38] was used. This is an observational tool consisting of 27 items that explore five specific areas: (1) child activity, (2) child emotional expressiveness, (3) child arousal state, (4) child vocalization, and (5) caregiver engagement. The total score ranged from 23.33 to 100 points, and higher scores corresponded to higher levels of distress. Since a validated Italian version of the scale was not available, the tool was administered only by English-speaking nurses, in line with Liguori et al [39].

#### Social Impact Indicators

This area was self-assessed by family caregivers at T3, by answering questions about aspects such as how often caregivers called the hospital for further information, experienced and managed complications at home after discharge (eg, child pain or bleeding), or accessed the pediatric emergency department of the hospital.

#### mHealth App Usage

Data relating to the use of the mHealth app by family caregivers in the EG (eg, number of app accesses, the number and type of documents consulted, time spent exploring the app) were collected.

#### mHealth App Satisfaction

A satisfaction survey regarding the app was administered at T3. Participants were asked (1) to rate the app’s completeness, usefulness, and ease of understanding; (2) to indicate whether they had sought additional information online due to dissatisfaction with the app’s content; (3) to report any technical issues experienced while using the app; and (4) to provide an overall satisfaction rating.

The STAI Forms Y-1 and Y-2, the HLS-EU-Q16, and the m-YPAS are validated instruments. However, the questionnaires administered online have not been specifically validated for online use.

The questionnaires administered at T0 and T2 were completed online in the ENT surgical department via a tablet provided by nurses to the EG and CG family caregivers. The T1 assessment was completed instead in paper form. Furthermore, the T3 evaluation was conducted online, after sending caregivers an email with a link to complete the relevant questionnaires. The latter approach was chosen since not all caregivers bring their children for follow-up at the MCHH promoting the study, sometimes preferring to refer and consult their general pediatrician.

Before the study began, nurses in the ENT surgical department involved in enrollment and subsequent measurements were adequately trained on the use of the assessment tools through the provision of standardized instructions. The training was conducted by a nurse from the research team and took place in a meeting room of the department.

### Sample Size

The sample size was estimated considering preoperative anxiety as the primary outcome. The sample size was estimated considering parental preoperative anxiety as the primary outcome, assessed using the Italian version of the State-Trait Anxiety Inventory (STAI-Y) [37]. The expected mean anxiety score, informed by data previously reported in an Italian pediatric surgical population [40], was 50 (SD 13) in the CG and 45 (SD 10) in the EG, corresponding to an effect size of 0.43. Based on these assumptions, a total sample of 180 participants was required, with 90 participants allocated to each group. The calculation was performed using G*Power for a 2-group Wilcoxon-Mann-Whitney test, assuming a significance level of 0.05 and a power of 80% (*β*=.20).

### Data Analysis

Description of the sample was based on frequency and percentage for categorical variables and on median and IQR for continuous variables. The standard statistical analysis was for intention-to-treat, and the balancing of baseline characteristics of the sample between the 2 groups of intervention was tested. The chi-square test or the exact Fisher test, as appropriate, was used to assess the association between 2 categorical variables. The nonparametric Wilcoxon-Mann-Whitney test was applied to evaluate the difference in the distribution of a continuous variable between 2 modalities of a categorical variable. The nonparametric Wilcoxon signed-rank test was calculated to test the difference between 2 paired continuous variables. Correlation was evaluated with the Spearman coefficient; a *P* value <.05 was considered statistically significant. SAS (version software 9.4 (SAS Institute Inc.) was used to conduct all statistical analyses. All randomized participants were analyzed in the group to which they had originally been assigned, regardless of intervention adherence. Reasons for study withdrawal were documented and recorded. Missing data were not imputed, and analyses were conducted using available data only. Data from the EG were analyzed in detail using the Wilcoxon rank-sum test and Spearman’s rank correlation coefficient.

### Ethical Considerations

This study received approval from the local ethics committee (CEUR-2022-Sper-26). As the intervention consisted exclusively of providing information and educational content through a smartphone app, it was intended as a low-risk intervention, and no serious adverse events were expected. Written informed consent was obtained from all caregivers through an offline, paper-based procedure, and no compensation was provided. Participants indicated their choice by selecting either a box confirming their willingness to participate or a box declining consent. Participants were informed that they could withdraw from the study at any time without the need to provide a reason. Children over 6 years of age were also asked to assent to participate in the study, signing a simplified, age-appropriate document that explained the study and what their agreement entailed. All personal data were processed in accordance with applicable data-protection regulations, and anonymization procedures were implemented prior to analysis.

## Results

### Participant Characteristics

**Figure 1.**
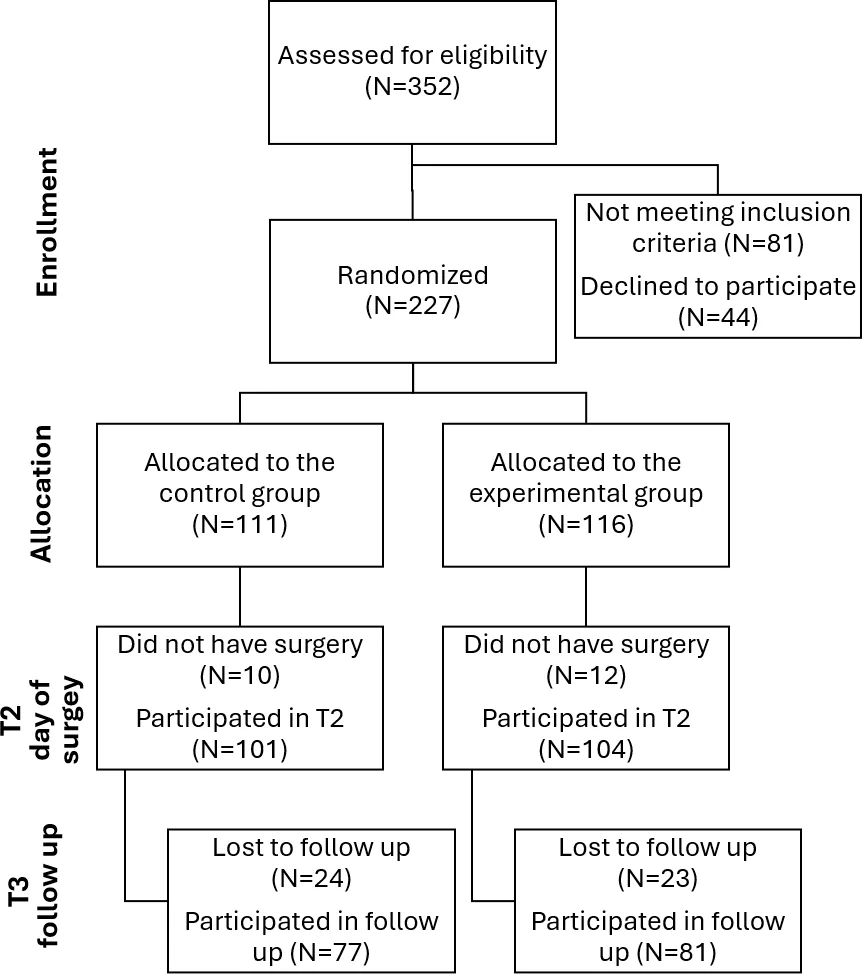
Participants’ flow diagram.

A total of 352 caregivers were screened between December 2022 and December 2024. Of these, 125 were excluded due to ineligibility or refusal to participate, resulting in the enrollment of 227 family caregivers of pediatric ENT patients, which met the target sample size. The participant flow diagram is detailed in Figure 1. As some questionnaires were not completed by all participants, only the available data were included in the analyses.

Participants were randomly assigned to either the EG (n=116) or the CG (n=111). In both groups, women represented the clear majority (CG: n=90, 82.57%; EG: n=92, 79.31%). Their educational backgrounds spanned a wide range, from lower-secondary to postgraduate levels, with upper-secondary schooling being the most common (CG: n=61, 55.96%; EG: n=59, 50.86%). The majority were employed (CG: n=90, 82.57%; EG: n=94, 81.03%), often in clerical or office-based roles (CG: n=50, 45.87%; EG: n=57, 49.14%), though various occupational categories were represented. Caregivers were generally in mid-adulthood, with most falling between 30 and 49 years of age. The most common surgical procedures among children in both groups were adenoidectomy alone and tonsillectomy combined with adenoidectomy, which together accounted for 82.56% (n=90) of surgeries in the CG and 80.17% (n=93) in the EG.

At baseline, caregivers showed a distribution of HL levels ranging from inadequate to sufficient, with the majority classified in the sufficient category (CG: n=69, 62.73%; EG: n=72, 62.07%). The initial anxiety median scores in the CG were 37 (IQR 30‐44) for the STAI-S and 36 (IQR 32‐43) for the STAI-T scores, while the initial anxiety median scores were 36 (IQR 31‐44) for the STAI-S and 36 (IQR 31‐42) for the STAI-T in the EG.

A complete set of participants’ characteristics is reported in Table 1.

**Table 1.** Baseline characteristics, health literacy (HL) levels and state (STAI Form Y-1 [STAI-S]) and trait (STAI Form Y-2 [STAI-T]) anxiety scores of family caregivers and surgical procedures by study group.

Characteristics	Control group (n=111)^a^	Experimental group (n=116)
Caregiver sex, n (%)		
Female	90 (82.57)	92 (79.31)
Male	19 (17.43)	24 (20.69)
Intersex	0 (0)	0 (0)
Caregiver country of birth, n (%)		
Italy	92 (84.40)	97 (83.62)
Foreign country	17 (15.60)	19 (16.38)
Caregiver native language, n (%)		
Italian	95 (87.16)	102 (87.93)
Non-Italian language	14 (12.84)	14 (12.07)
Caregiver highest educational attainment, n (%)		
Primary education	0 (0.00)	1 (0.86)
Lower secondary education	13 (11.93)	13 (11.21)
Upper secondary education	61 (55.96)	59 (50.86)
Vocational education	1 (0.92)	0 (0.00)
University degree (bachelor’s or master’s degree)	24 (22.02)	33 (28.45)
Postgraduate education	9 (8.26)	10 (8.62)
Other	1 (0.92)	0 (0.00)
Employment status, n (%)		
Unemployed	19 (17.43)	22 (18.97)
Employed	90 (82.57)	94 (81.03)
Current occupation, n (%)		
Manager or entrepreneur	6 (5.50)	10 (8.62)
Clerical or office worker	50 (45.87)	57 (49.14)
Self-employed professional	8 (7.34)	11 (9.48)
Manual laborer	26 (23.85)	16 (13.79)
Unemployed	19 (17.43)	22 (18.97)
Caregiver age (y), n (%)		
<30	5 (4.63)	6 (5.17)
30‐39	48 (44.44)	47 (40.52)
40‐49	51 (47.22)	54 (46.55)
≥50	4 (3.70)	9 (7.76)
Surgical procedures, n (%)		
Adenoidectomy alone	45 (41.28)	36 (31.03)
Tonsillectomy alone	10 (9.17)	14 (12.07)
Adenoidectomy + tympanostomy tube placement	4 (3.67)	9 (7.76)
Tonsillectomy + adenoidectomy	45 (41.28)	57 (49.14)
Tonsillectomy + tympanostomy tube placement	1 (0.92)	0 (0.00)
Combined adenoidectomy, tonsillectomy, and tympanostomy tube placement	4 (3.67)	0 (0.00)
Children gender, n (%)		
Female	44 (40)	47 (41)
Male	65 (60)	69 (59)
Children age, median (IQR)	5 (4-6)	6 (4-7)
T0 HL scores, n (%)		
Inadequate	7 (6.36)	11 (9.48)
Problematic	34 (30.91)	33 (28.45)
Sufficient	69 (62.73)	72 (62.07)
Score T0 STAI-S, median (IQR)	37 (30‐44)	36 (31‐44)
Score STAI-T, median (IQR)	36 (32‐43)	36 (31‐42)

a Some questionnaires were not completed by all participants. In the control group, 3 sociodemographic questionnaires and 1 STAI Form Y-2 or State-Trait Anxiety Inventory questionnaire were not submitted. Furthermore, 1 control group participant did not provide a valid age value.

### Caregiver Anxiety (Distribution of STAI Scores)

Total STAI-S scores changed significantly over time (*P<.*0001). The median score increased from 36 (IQR 31‐42) at T0 to 38 (IQR 33‐46) at T2, indicating a temporary rise in anxiety following baseline, since at T3, the median score decreased to 32 (IQR 28‐39), reflecting a longer-term reduction in anxiety. All changes were statistically significant (*P<.*0001).

No difference in the distribution of caregiver state anxiety was found between T2 and T0 when comparing the EG and CG. The same result emerged between T3 and T0 and between T3 and T2. Table 2 presents the distribution of changes in caregivers’ state anxiety levels (STAI-S) at the different measurement time points by study group.

**Table 2.** Distribution of the difference in the caregiver state anxiety levels (STAI Form Y-1 [STAI-S]) between T0, T2, and T3 by study group^a^.

Difference in STAI-S between T0, T2, and T3	CG^b^		EG^c^		
	Participants, n	Median (IQR; min-max)	Participants, n	Median (IQR; min-max)	*P* value
STAI-S at T2—STAI-S at T0	98	2 (−2 to 7; −19 to 22)	96	3 (−1 to 7; −24 to 25)	.38
STAI-S at T3—STAI-S at T0	77	−2 (−11 to 1; −33 to 14)	81	−1 (−8 to 3; −25 to 24)	.08
STAI-S at T3—STAI-S at T2	75	−5 (−15 to 0; −43 to 12)	76	−6 (−12 to 0; −35 to 23)	.82

a Group differences were assessed using the nonparametric Wilcoxon Mann-Whitney test. Statistical significance was defined as *P<.*05.

b CG: control group.

c EG: experimental group.

From T0 to T2, both the CG and the EG showed a statistically significant increase in anxiety. In the CG, the median change was 2 (IQR −2 to 7), while in the EG it was 3 (IQR −1 to 7). From T0 to T3, the CG showed a median decrease of –2 (IQR −11 to 1), and the EG reported a median change of –1 (IQR −8 to 3). Nonetheless, the EG reported significantly more favorable scores than the CG for the statement “I feel comfortable” (item 10) of the STAI-S at T2 (*P*=.04; Table 3).

**Table 3.** Distribution of responses of item 10 of STAI Form Y-1 (STAI-S) at T2.

STAI-S at T2	CG^a^, n (%)	EG^b^, n (%)	*P* value
Item 10: “I feel confident”			.04
1	17 (17.35)	12 (12.50)	
2	60 (61.22)	57 (59.38)	
3	13 (13.27)	25 (26.04)	
4	8 (8.16)	2 (2.08)	

a CG: control group.

b EG: experimental group.

### Caregiver’s Preparation for Hospital Admission and Surgery

The data collected at T1 were lost due to the renovation of the hospital’s administrative Welcome Point, which resulted in the displacement of the stored materials.

### Child Distress (M-YPAS)

No statistical difference in child distress m-YPAS score at T2 emerged between the groups (*P=*.10), with equal median values (CG: 23.40; EG: 23.40) and overlapping IQRs (CG: 23.40‐38.40; EG: 23.40‐29.20).

### Child’s Preparation for Surgery

Both groups demonstrated consistently high compliance with preoperative instructions, such as arriving fasting (CG: n=97, 100%; EG: n=97, 99.0%), maintaining hygiene (100% in both groups, n=97 and n=98, respectively), and removing nail polish (CG: n=97, 100%; EG: n=93, 94.9%) or jewelry (CG: n=95, 97.9%; EG: n=97, 99.0%). Overall, no statistically significant differences were observed between the CG and EG regarding any aspect of the child’s preparation for surgery (all *Ps<.*05).

### Social Impact Indicators

Most social impact indicators did not show significant differences between the CG and EG. For the item “On a scale from 1 to 10, how adequate was the in-person time allocated by the medical and nursing staff at the MCHH throughout your child’s surgical care pathway?”, however, a significant difference emerged in the score distribution (*P=.*03). A larger proportion of parents in the CG assigned the maximum score of 10 (n=30, 39.0% vs n=17, 21% for the CG and EG, respectively), whereas the EG ratings were more concentrated around intermediate values (6‐7: n=8, 10.4% vs n=21, 25.9% for the CG and EG, respectively).

### mHealth App Usage and Satisfaction (EG Only)

The median duration of app use in the EG was 6 (IQR 5‐7) days. Overall, 87 (75%) out of 116 caregivers in the EG viewed at least 1 content item, with a minimum of 13 contents opened and a maximum of 120. In particular, 35.3% (n=41) viewed fewer than 5 items. The 10 topics that attracted the most caregivers were, in decreasing order of number of views, the following: “Postoperative pain management (n=171),” “Postoperative diet at home (n=161),” “Postoperative home-care instructions (n=125),” “Items to bring to the hospital on the day of admission and surgery (n=117),” “How to prepare my child for outpatient visits, hospital admission, and surgery (n=102),” “The operating room (n=102),” “Can my child eat whatever they want? (n=102),” “From the ward to the operating room (n=100),” “On the ward after surgery (n=89),” and “Can my child bathe/wash? (n=87).”

A detailed analysis of the data from the EG, conducted using the Wilcoxon rank-sum test and Spearman’s rank correlation coefficient, did not reveal a significant association between HL score and the number of viewed contexts (ρ=0.13, 95% CI –0.05 to 0.31; *P=*.16). A significant negative correlation emerged between the number of app contents opened and both the mYPAS child distress score (ρ=–0.23, 95% CI –0.40 to –0.04; *P*=.02) and the STAI-S caregiver state anxiety score at T0 (ρ=–0.22, 95% CI –0.39 to –0.04; *P*=.02), indicating that higher engagement with app contents was associated with lower child distress at T2 and with lower caregiver state anxiety levels at T0 in the EG, respectively.

The app was evaluated through a satisfaction questionnaire completed by 46.55% (n=56) of the users. Overall, respondents rated the app as “very” or “completely” complete (n=46, 85.18%), useful (n=48, 88.89%), and easy to understand (n=29, 53%). Most participants reported “never” having searched for additional information online due to dissatisfaction with the app’s content (n=36, 66.67%) and indicated that they did not experience technical issues while using the app (n=47, 87.04%). Regarding overall satisfaction, the majority of users were “completely” (n=14, 25.93%) or “very” (n=26, 48.15%) satisfied with the app. Users’ overall satisfaction was not significantly associated with baseline characteristics, HLS-EU-Q16, STAI, or m-YPAS scores.

## Discussion

### Principal Findings

This RCT evaluated whether a co-designed mHealth app could reduce caregiver anxiety during the pediatric ENT perioperative pathway. Contrary to our hypothesis, no significant differences in anxiety were found between caregivers who received the mHealth app in addition to standard care and those who received standard care alone. Likewise, no significant between-group differences were observed for child distress, child preparation for hospitalization and surgery, and postoperative management at home. This lack of detectable effects of using the mHealth app may appear disappointing, yet it aligns with a growing body of evidence showing limited effectiveness of app-based interventions in pediatric perioperative care, particularly regarding caregiver anxiety [15]. Overall, while mHealth tools have shown promise in reducing distress among children [15,41], evidence of benefits for caregivers’ anxiety remains weak [15,42]. In this light, the null findings reinforce the need for critical reflection on both methodological and contextual factors that may have influenced the effectiveness of the intervention. First, even if the baseline characteristics of the CG and EG overlapped, over one-third of the sample of family caregivers in the EG had a low HL level**,** which may have reduced their ability to understand, appraise, or use the health information provided through the app [18]. Moreover, although the language of the content was simplified, 1 in 6 of caregivers in the EG spoke a different native language. Therefore, the extent to which linguistic or literacy-related barriers influenced the EG’s ability to use the app remains unknown [43].

A further factor that may have influenced the findings is that participants in the CG may have independently sought information online, consistent with the phenomenon known as “contamination by informal information-seeking,” which is now well documented in the literature [44], thereby blurring the differences between the 2 groups.

Another contributing factor may relate to the timing of anxiety measurement, which occurred shortly before surgery, a moment that may reflect an expected physiological peak in parental stress rather than a modifiable psychological state [40]. This calls into question whether state anxiety is the most sensitive or appropriate outcome to capture the effects of digital tools that may operate more subtly, by improving knowledge, emotional preparedness, or perceived control. Notably, anxiety levels increased for both groups between baseline and the day of surgery, reflecting expected surgery anticipatory stress [44]. Following surgery, anxiety levels decreased in both groups, consistent with previous findings [41]. This pattern suggests that perioperative anxiety may evolve over time according to the natural course, regardless of the specific support tool used.

Finally, the time available to engage caregivers in using the app before surgery may have been insufficient. With a median usage period of only 6 days, caregivers may have had limited opportunities to explore and interact with the tool and related content and functionalities, given competing responsibilities and the demands of daily life.

Regarding app use, a notable share of caregivers either did not access the app or used it only minimally. Several factors may account for this limited use. One plausible factor is variation in individual preferences for how educational content was delivered. A systematic review of pediatric pretonsillectomy education programs found that interventions using smartphone apps, brochures, text messages, and videos all showed variable levels of engagement and efficacy, depending on the modality used and the target audience selected, suggesting that engagement may be influenced more by preferred learning styles than content quality alone [13]. Consistent with previous literature, another contributing factor may have been that some caregivers preferred direct human interaction and information delivered by health care professionals [45,46]. This interpretation is also supported by the findings on social impact indicators, as only 1 item showed a significant difference between the groups: parents in the CG rated the adequacy of in-person time provided by staff more highly than those in the EG. This pattern may suggest that reliance on face-to-face communication increased the perceived value of direct interaction, whereas access to the app may have shifted expectations toward digital support, influencing perceptions of in-person contact rather than actual staff time. This interpretation is consistent with evidence that technology-based preparatory tools can modify caregivers’ expectations about interpersonal communication and shape how they appraise and value face-to-face interactions [47].

Another barrier to app use may have been that some family caregivers perceived it as difficult to navigate and engage with it because of the volume and density of the content provided, which may have led to information overload [47]. Although overall satisfaction with the app was high among approximately half of the participants who completed the app satisfaction evaluation survey, our results show that caregivers with higher baseline anxiety were less likely to engage with the app. This finding is consistent with Zhuo et al [48], who distinguish “monitors” (active seekers) from “blunters” or “avoiders.” These different information-seeking behaviors suggest that those experiencing higher anxiety at preadmission, and who presumably would have needed the most support in managing it, were less inclined to use the app. Such behavioral heterogeneity, typical in nonstratified samples, may have diluted observable group differences.

Usage patterns also led researchers to hypothesize that usability barriers may have emerged from the document format: materials were indeed provided as PDFs and did not adapt to screen size, resulting in a text font size that was too small to read, necessitating frequent zooming and panning on mobile devices.

Despite the general null findings, a dose-response effect was observed in the EG: caregivers who accessed more than 5 pieces of content had children who appeared less stressed preoperatively, supporting the findings of Wang et al [44].

Moreover, a slightly higher level of preoperative comfort was found in the EG. This small advantage may reflect the reassuring effect of clearer procedural and orientation information provided through the app, consistent with evidence that preoperative education can reduce parental anxiety [28].

Beyond the specific findings of this study, it is important to acknowledge that null results can provide valuable contributions to the evidence base. Reporting studies that do not demonstrate significant effects helps prevent publication bias and offers a more balanced understanding of the effectiveness of digital health interventions [49,50]. Such results can also inform the refinement of app design and implementation strategies, as well as help recalibrate unrealistic expectations regarding the potential impact of digital health solutions in complex clinical contexts. More broadly, making these findings available supports research transparency, improves the accuracy of future systematic reviews and meta-analyses, and ultimately contributes to cumulative scientific progress [49,50].

### Limitations

This study has several limitations. First, caregivers and nurses were not blinded to group allocation, which may have introduced expectation- and assessment-related biases, respectively. Furthermore, the RCT findings may have been affected by contamination bias resulting from caregivers’ informal information-seeking from multiple additional sources [25], including health care professionals, printed materials, websites, and social media. Future studies could assess information-seeking behaviors more systematically (and stratify the sample accordingly) to explore their influence on intervention effectiveness [48].

Another limitation concerns the variability in engagement with the intervention. Many caregivers only accessed a limited portion of the app content and therefore did not receive the intended intervention “dose”, which may have affected the ability to detect significant effects. Future studies should explore alternative and more engaging content delivery formats, such as videos, podcasts, and gamification features (eg, feedback, challenges, or rewards), which may enhance accessibility, usability, and user engagement with digital interventions [51,52].

Moreover, caregivers’ reading fluency and comprehension competencies as well as document or digital literacy were not assessed in this study, although these skills affect the ability to navigate written materials, particularly when delivered in PDF files that are not optimized for mobile devices.

Furthermore, the timing of the measurement of anxiety, taken shortly before surgery, is another limitation, as it coincides with an expected peak of anticipatory stress. This may have reduced the sensitivity for detecting short-term changes attributable to app use.

Moreover, the app’s navigability and information architecture, such as the length or text density of written materials, may not have fully supported ease of use for all users. These considerations are addressed in the Guide to Implementing the Health Literacy Universal Precautions Toolkit and its communication-design guidance, although this resource was not incorporated into the app’s design and development process [53].

A further limitation was the loss of data collected at T1 regarding caregivers’ preparation for hospital admission. However, this did not affect the study’s primary or key secondary outcomes.

Finally, the single-site study design limits the generalizability of the findings, as participants may not be representative of broader linguistic, cultural, or sociodemographic populations.

### Conclusions

The findings of this RCT suggest that adding an mHealth app to standard care did not significantly reduce caregiver anxiety. Beyond its clinical outcomes, this study provides a methodological contribution through the development and rigorous evaluation of a user-centered mHealth intervention for family caregivers of pediatric surgical patients. The trial also underscores key challenges associated with the implementation of digital health interventions in real-world clinical settings, including variability in user engagement, the timing and duration of intervention exposure, and the potential influence of information-seeking behaviors. These factors should be carefully considered when interpreting intervention effectiveness and when designing future trials.

Rather than questioning the value of mHealth interventions, these findings highlight that future research should shift its focus from developing and evaluating digital tools alone to understanding how they can be effectively implemented, integrated into routine care, and how engagement with digital interventions can be optimized across the perioperative pathway. This will require implementation strategies that promote meaningful and sustained engagement, while identifying which caregivers are most likely to benefit from mHealth-supported education. Optimizing caregiver support throughout the perioperative pathway remains an important objective, given its potential to positively influence the perioperative experience of both caregivers and children.

## Supplementary material

10.2196/93989 Checklist 1 CONSORT-EHEALTH (version 1.6) Checklist.

## Abbreviations

CG: control group
CONSORT-EHEALTH: Consolidated Standards of Reporting Trials of Electronic and Mobile Health Applications and Online Telehealth
EG: experimental group
ENT: ear, nose, and throat
HL: health literacy
HLS-EU-Q16: 16-item European Health Literacy Survey Questionnaire
ISR: Information System Research
m-YPAS: modified version of the Yale Preoperative Anxiety Scale
MCHH: maternal and child health hospital
mHealth: mobile health
RCT: randomized controlled trial
STAI-S: STAI Form Y-1
STAI-T: STAI Form Y-2
STAI-Y: State-Trait Anxiety Inventory
